# Impaired theta-gamma coupling in APP-deficient mice

**DOI:** 10.1038/srep21948

**Published:** 2016-02-24

**Authors:** Xiaomin Zhang, Wewei Zhong, Jurij Brankačk, Sascha W. Weyer, Ulrike C. Müller, Adriano B. L. Tort, Andreas Draguhn

**Affiliations:** 1 Institute for Physiology and Pathophysiology, Heidelberg University, Heidelberg, Germany; 2 Institute of Pharmacy and Molecular Biotechnology, Department of Bioinformatics and Functional Genomics, Heidelberg University, Heidelberg, Germany; 3 Brain Institute, Federal University of Rio Grande do Norte, Natal, Brazil

## Abstract

Amyloid precursor protein (APP) is critically involved in the pathophysiology of Alzheimer’s disease, but its physiological functions remain elusive. Importantly, APP knockout (APP-KO) mice exhibit cognitive deficits, suggesting that APP plays a role at the neuronal network level. To investigate this possibility, we recorded local field potentials (LFPs) from the posterior parietal cortex, dorsal hippocampus and lateral prefrontal cortex of freely moving APP-KO mice. Spectral analyses showed that network oscillations within the theta- and gamma-frequency bands were not different between APP-KO and wild-type mice. Surprisingly, however, while gamma amplitude coupled to theta phase in all recorded regions of wild-type animals, in APP-KO mice theta-gamma coupling was strongly diminished in recordings from the parietal cortex and hippocampus, but not in LFPs recorded from the prefrontal cortex. Thus, lack of APP reduces oscillatory coupling in LFP recordings from specific brain regions, despite not affecting the amplitude of the oscillations. Together, our findings reveal reduced cross-frequency coupling as a functional marker of APP deficiency at the network level.

The accumulation of amyloid plaques derived by proteolytic cleavage of APP is one of the leading hallmarks of Alzheimer’s disease (AD). Overexpression of APP elicits AD-like abnormalities, such as neuronal degeneration and memory impairment^1^,^2^. Besides its role in pathological processes, however, APP is involved in normal physiological processes, such as neuronal morphogenesis and maintenance of synaptic transmission and plasticity, probably by mediating neuronal adhesion and secretion of the soluble ectodomain sAPPα^3^,^4^,^5^. In accordance, hippocampal CA1 neurons of mice lacking APP (APP-KO mice) have decreased spine density and dendritic complexity^6^,^7^. Aged APP-KO mice also have impaired long-term potentiation (LTP), and, behaviorally, exhibit deficits in the Morris water maze as well as in passive avoidance learning^8^,^9^. Taken together, these results indicate that APP-KO mice provide a unique opportunity to investigate how APP affects network activity.

Coherent network activity forms a crucial interface between cellular and cognitive functions. Abnormal brain oscillations have been proposed as functional biomarkers of many neuropsychiatric disorders^10^,^11^,^12^,^13^. In particular, AD patients may have increased theta along with decreased gamma amplitude^14^,^15^,^16^. Brain oscillations of different frequencies are not isolated entities; in fact, theta and gamma oscillations not only co-occur but also interact with each other, a phenomenon called cross-frequency coupling (CFC)^17^. Functional roles of CFC between theta phase and gamma amplitude have been suggested^17^,^18^. For instance, theta-gamma coupling correlates with performance in learning tasks^19^, may facilitate inter-area communication^20^,^21^, and would constitute a memory buffer^22^. Recent work on a mouse model of AD showed that impaired theta-gamma coupling arises before Aβ accumulation^23^, suggesting that altered CFC may represent an early biomarker for AD.

In the present work we investigated whether normal APP function is important for different network oscillations and their interactions. To that end, we recorded local field potentials (LFPs) in the posterior parietal cortex, dorsal hippocampus and lateral prefrontal cortex (PFC) of freely behaving wild-type and APP-KO mice. Our results reveal that lack of APP impairs CFC despite not affecting the power of individual frequency bands. This effect may be of importance for the cognitive deficits observed in APP-KO mice, and it may prove useful for developing non-invasive biomarkers of neurodegenerative diseases.

## Results

### Lack of APP leads to impaired cross-frequency coupling in recordings from the parietal cortex

**Figure 1:**
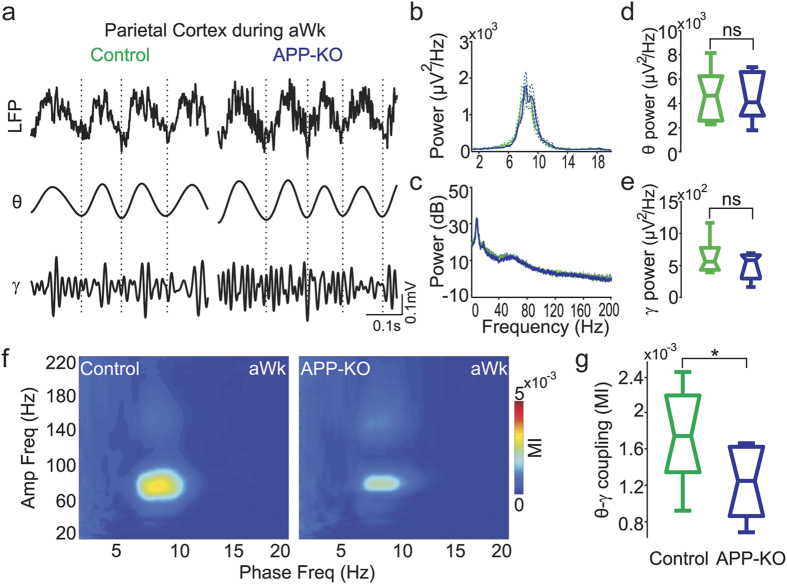
Impaired theta-gamma coupling during active waking in recordings from the posterior parietal cortex of APP-KO mice. (**a**) Representative example of raw and filtered recordings from the posterior parietal cortex during active waking (aWk) of control and APP-KO mice. (**b**) Mean power spectra from 1 to 20 Hz of control (green) and APP-KO mice (blue). Dashed lines indicate ± SEM (n = 9 mice per group). (**c**) Mean power spectra from 1 to 200 Hz in dB scale. (**d,e**) Boxplot distribution of mean theta (**d**) and gamma (**e**) band power. Notice no difference between control and APP-KO mice. (**f**) Mean comodulation map of control and APP-KO mice during aWk. Pseudocolor scale indicates the modulation index (MI, see Methods). (**g**) Mean theta-gamma MI in control and APP-KO mice. Coupling strength is significantly decreased in APP-KO mice (*p < 0.05).

The posterior parietal cortex is crucial for spatial memory formation^24^,^25^. This neocortical region integrates visual and self-motion information at a relatively early stage of perceptual processing, and sends egocentric information into the hippocampus where it is transformed into allocentric information^25^. To investigate whether APP influences parietal networks, we performed surface recordings in aged APP-KO and control mice (n = 9 for each group). Prominent theta (4–12 Hz) and gamma (30–80 Hz) oscillations were observed in both control and APP-KO mice during active waking (aWk) (Fig. 1a), with similar power levels in both groups (Fig. 1b–e). Moreover, theta and gamma peak frequency were also unaltered in APP-KO mice (Fig. 1b,c).

Accumulating evidence indicates that the coupling between the phase of a slow oscillation and the amplitude of faster oscillations may be involved in information processing^17^,^18^,^19^. We next measured phase-amplitude CFC during aWk and found strong theta-gamma coupling in wild type (control) mice (Fig. 1f, left panel), which is consistent with previous reports^26^,^27^,^28^. Intriguingly, however, despite the normal power levels of the individual oscillations (Fig. 1b–e), parietal theta-gamma coupling was much weaker in APP-KO mice (Fig. 1f,g; p = 0.019).

**Figure 2:**
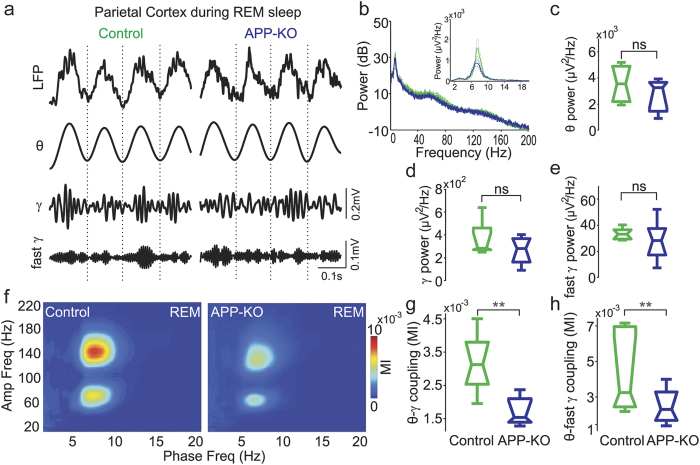
Lack of APP also affects theta-fast gamma coupling in parietal recordings during REM sleep. (**a**) Representative example of raw and filtered recordings from the posterior parietal cortex during REM sleep of control and APP-KO mice. (**b**) Mean power spectra from 1 to 200 Hz in dB scale (control: green; APP-KO: blue). Inset shows mean power spectra from 1–20 Hz. Dashed lines indicate ± SEM (n = 9 mice per group) (**c–e**) Boxplot distribution of mean theta (**c**), gamma (**d**) and fast gamma (**e**) band power. Notice similar power levels in control and APP-KO mice for all oscillations. (**f**) Mean comodulation map of control and APP-KO mice during REM sleep. (**g,h**) Mean theta-gamma (**g**) and theta-fast gamma (**h**) MI. Coupling strength between theta phase and the amplitude of both gamma and fast gamma is significantly decreased in APP-KO mice (**p < 0.01).

While aWk is crucial for memory encoding, REM sleep is believed to be important for memory consolidation^29^,^30^,^31^. In this state, fast gamma (120–160 Hz) oscillations also appear in the power spectrum^26^. We found no differences between APP-KO and control animals regarding band power or peak frequencies of theta and gamma activities during REM (Fig. 2b–e). Similarly to previous work^26^,^27^,^28^, during REM sleep we found that theta phase modulated not only gamma but also fast gamma oscillations in recordings from the posterior parietal cortex of wild-type mice (Fig. 2f). As in aWk, APP-KO mice exhibited significantly weaker theta-gamma coupling than control animals during REM (Fig. 2g; p < 0.01). In addition, theta-fast gamma coupling was also significantly decreased (Fig. 2h; p < 0.01). Taken together, our results show that lack of APP leads to impaired coupling between theta and faster oscillations in recordings from the posterior parietal cortex during aWk and REM sleep, without causing major changes in power and peak frequency.

### Impaired cross-frequency coupling in recordings from the hippocampus of APP-KO mice

**Figure 3:**
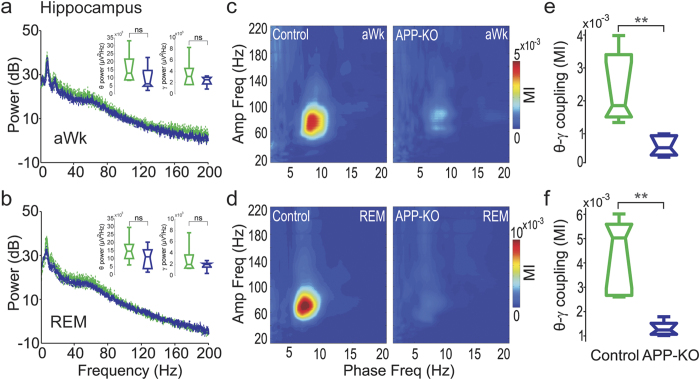
Impaired CFC in LFP recordings from the hippocampus of APP-KO mice. (**a,b**) Mean hippocampal power spectra during aWk (**a**) and REM sleep (**b**) for control (green) and APP-KO (blue) mice. Theta and gamma band power levels were not different between control and APP-KO mice (see inset panels for boxplot distributions). (**c,d**) Mean comodulation map of control and APP-KO mice during aWk (**c**) and REM sleep (**d**). (**e,f**) Mean theta-gamma MI during aWk (**e**) and REM sleep (**f**). APP-KO mice have significantly decreased coupling strength in both states (**p < 0.01).

Previous work has shown that aged APP-KO mice have impaired LTP in the hippocampal CA1 region and poor performance in the Morris water maze task^8^,^9^,^32^, a hippocampal-dependent paradigm for studying spatial memory^33^. This suggests that mice deficient for APP may have altered network activity in the hippocampus. To address this question, we performed LFP recordings in stratum radiatum and stratum oriens of the dorsal CA1 region during aWk and REM sleep (Supplementary Fig. S1). We observed layer- and state-dependent differences of CFC patterns in both genotypes. During aWk, theta-gamma coupling strength gradually decreased from stratum radiatum to stratum oriens, in line with previous findings^13^,^34^,^35^. During REM sleep, theta-gamma coupling could be observed in stratum radiatum (Fig. 3), whereas theta-gamma and theta-fast gamma coupling coexisted in stratum oriens, resembling the CFC pattern observed in the posterior parietal cortex (data not shown, but see refs 34 and 35). As in recordings from the parietal cortex, we did not observe statistically significant differences in power and peak frequency of hippocampal theta and gamma oscillations between control and APP-KO mice during both behavioral states (see Fig. 3a,b, data from stratum radiatum). However, the strong coupling between theta and gamma observed in control mice nearly vanished in APP-KO mice in both aWk and REM states (Fig. 3c–f; p < 0.01). Similar results were obtained for stratum oriens (not shown). Thus, our data indicate that APP is necessary for hippocampal theta-gamma coupling.

### Normal cross-frequency coupling in recordings from the prefrontal cortex of APP-KO mice

**Figure 4:**
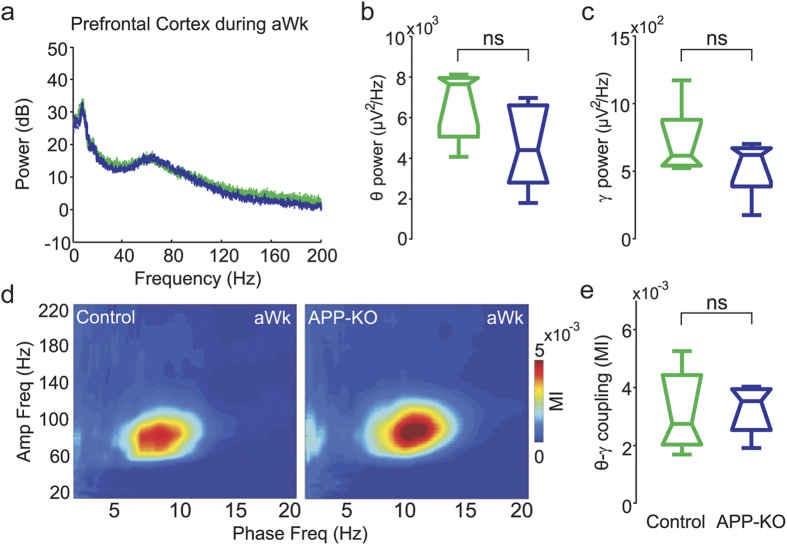
Normal cross-frequency coupling during active waking in recordings from the prefrontal cortex of APP-KO mice. (**a–e**) Shown are mean power spectra (**a**), along with mean theta (**b**) and gamma (**c**) band power, mean comodulation map (**d**), and mean theta-gamma coupling strength (**e**) for PFC recordings during aWk in control (green) and APP-KO (blue) mice. Notice no differences in power content and CFC levels between genotypes.

The lateral PFC is involved in working memory and decision making^36^,^37^. To address whether APP affects network activity in this region, we evaluated the spectral content of PFC LFPs as well as CFC in control and APP-KO mice during aWk and REM. We found that the peak frequency and band power of neither theta nor gamma differed between genotypes during aWk (Fig. 4a–c). Comodulation maps showed strong theta-gamma coupling during aWK in both control and APP-KO mice (Fig. 4d), without difference in coupling strength between both genotypes (Fig. 4e). Likewise, during REM sleep the peak frequency and band power of theta and gamma oscillations were not different in control and APP-KO mice (Supplementary Fig. S2). In contrast to aWk, during REM sleep the coupling of theta and gamma was barely visible in both genotypes, probably due to the low gamma activity in the lateral PFC during REM sleep (as judged by the power spectrum; Supplementary Fig. S2). Thus, contrary to the parietal cortex and hippocampus, APP-KO mice have normal theta-gamma coupling in recordings from the PFC. Therefore, the impairment of oscillatory coupling associated with the lack of APP is not global but rather depends on the recorded region.

### Lack of APP does not affect the occurrence of gamma bursts nor the theta phase of maximal gamma amplitude

**Figure 5:**
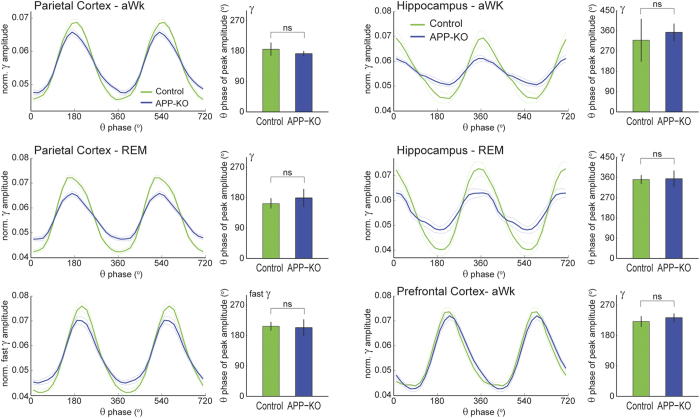
Lack of APP does not affect the theta phase of maximal gamma amplitude. The line plots show the mean (fast) gamma amplitude as a function of theta phase for control (green) and APP-KO (blue) mice; the results were obtained from the same data as in Figs 1, 2, 3, 4, as labeled (hippocampal recordings correspond to stratum radiatum below the reversal of theta phase). The amplitude was normalized within animals such that the sum of its values across a theta cycle is 1. The bar graphs show the mean theta phase of maximal amplitude in each case; error bars denote ± angular SD. Notice reduced variations of (fast) gamma amplitude within theta cycles in the parietal cortex and hippocampus of APP-KO animals, but not in prefrontal cortex. The theta phase of maximal amplitude is not different between genotypes.

The assessment of theta-gamma phase-amplitude coupling requires a long enough epoch to average out fluctuations in gamma amplitude that are not systematically coupled to the theta cycle (typically > 10–30 s; see ref. 47 for a review). In other words, CFC metrics inherently suffer from a poor time resolution; for instance, it is not possible to (statistically) assess the existence of theta-gamma coupling in individual theta cycles. Therefore, it remains to be determined whether the reduced theta-(fast) gamma coupling in recordings from the parietal cortex and hippocampus of APP-KO mice is due to lower variations of gamma amplitude within theta cycles or due to lower occurrence of theta cycles with nested gamma. To gain insight into this question, we next compared the amount of “gamma bursts” between control and APP-KO animals. An oscillatory burst was defined as the crossing of the instantaneous gamma amplitude of a threshold of 2SD above the mean amplitude. During aWk, we found that gamma bursts occurred at comparable rates between genotypes both in recordings from the hippocampus (control mice: 3.61 ± 0.08 Hz; APP-KO mice: 3.63 ± 0.11 Hz) and from the parietal cortex (control mice: 3.83 ± 0.09 Hz; APP-KO mice: 3.75 ± 0.10 Hz). Similar results were found during REM sleep for gamma and fast gamma (not shown). Interestingly, further analyses revealed that lack of APP also did not affect the theta phase of maximal gamma amplitude in any of the recorded regions in either aWk or REM sleep (Fig. 5). These results suggest that APP controls the variations of gamma amplitude within theta cycles, but it does not influence the amount of oscillatory bursts nor the theta phase where the bursts occur.

### Impaired cross-regional coupling in APP-KO mice

**Figure 6:**
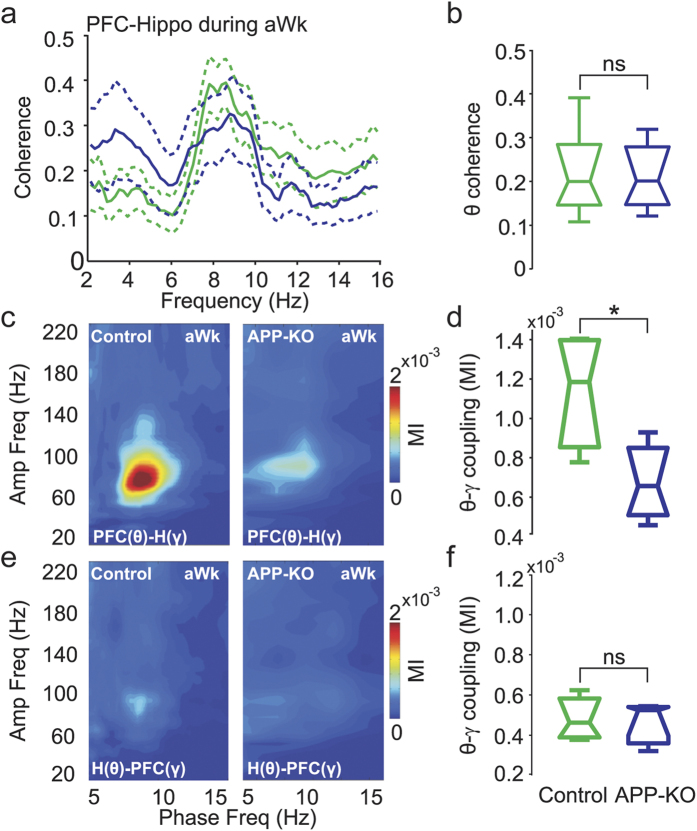
Impaired coupling between LFPs recorded in the dorsal hippocampus and in the lateral prefrontal cortex of APP-KO mice. (**a**) Phase coherence spectra between hippocampal and PFC recordings during aWk for control (green) and APP-KO (blue) mice. (**b**) Mean coherence in the theta band. Theta coherence is not different between control and APP-KO mice. (**c**) Mean cross-regional comodulation map obtained during aWk using the instantaneous theta phase in the PFC and gamma amplitude in the hippocampus (PFC(θ)-H(γ)). (**d**) Mean PFC(θ)-H(γ) MI, showing impaired cross-regional coupling in APP-KO mice (*p < 0.05). (**e**) Mean comodulation map for theta phase recorded in the hippocampus and gamma amplitude in the PFC (H(θ)-PFC(γ)). (F) Mean H(θ)-PFC(γ) MI. Notice low CFC levels in both genotypes.

Effective communication between the hippocampus and the neocortex is believed to depend on theta and gamma oscillations; for example, theta coherence between the hippocampus and prefrontal cortex increases during spatial task learning^38^,^39^. Moreover, PFC neurons and local gamma oscillations phase-lock to the hippocampal theta rhythm^40^. We next investigated whether APP plays a role in cross-regional coupling of prefrontal and hippocampal LFPs. Phase coherence spectra between the hippocampus and lateral PFC during aWk showed that neither theta (Fig. 6a,b) nor gamma coherence (not shown) is significantly changed in APP-KO mice. Comodulation maps obtained during aWk using the instantaneous phase time series in the lateral PFC and the instantaneous amplitude in the dorsal hippocampus revealed strong theta-gamma coupling in control mice (Fig. 6c). This cross-regional CFC was largely disrupted in APP-KO mice (Fig. 6c,d; p < 0.05). In contrast, gamma amplitude in the lateral PFC was only weakly modulated by the phase of hippocampal theta during aWk in both control and APP-KO mice (Fig. 6e,f). Similar findings were obtained for cross-regional analyses during REM sleep (Supplementary Fig. S3). In summary, our results reveal that APP-KO mice have impaired CFC in LFPs recorded within a brain region and also between LFPs recorded from different regions, despite having normal power levels in all recorded regions.

## Discussion

In this study we investigated the role of APP in coherent network activity, as assessed by oscillations in LFP recordings. We found that lack of APP is associated with normal peak frequency and band power of theta, gamma and fast gamma oscillations. However, mice deficient for APP had impaired interaction between slow and fast LFP rhythms: coupling between theta phase and gamma amplitude was significantly diminished both in recordings performed in the posterior parietal cortex and in the dorsal hippocampus, but, interestingly, not in recordings from the lateral prefrontal cortex. We also found decreased phase-amplitude coupling between recordings performed in different brain regions. Together, our findings suggest that impaired CFC is a functional marker of APP deficiency at the network level.

The proteolysis of APP leads to the accumulation of Aβ peptides, which is believed to be involved in the genesis of AD^1^,^2^. Despite its pathophysiological role, the main physiological functions of APP are currently unknown. Understanding these may shed new light on AD symptoms. Among potential functions, APP has been shown to have trophic effects; for instance, APP promotes neurite outgrowth and promotes synaptogenesis^41^. Moreover, dysfunction of APP has been associated with poorly formed synapses and deficits in neuronal signaling^6^,^7^,^8^. Behaviorally, exogenous administration of APP leads to memory enhancement^42^, and antibodies against APP impairs memory consolidation and retrieval^43^,^44^. In a parallel literature, network level analyses have been shown that brain oscillations and their interactions play a role in cognitive functions^45^,^46^. In particular, theta-gamma phase-amplitude coupling has been observed in multiple brain regions of several species^17^,^47^, and hypothesized to contribute to memory processes and coordinate communication between brain regions^17^. Therefore, the disrupted CFC in APP-KO mice observed here may constitute a network correlate of dysfunctional signaling and cognitive deficits that have been previously associated to the lack of APP^9^.

In control animals, we found prominent theta-gamma coupling in recordings from the parietal cortex during aWk as well as REM sleep. Theta-fast gamma coupling also hallmarked network activity in recordings from the parietal cortex, but this CFC only became prominent during REM sleep^26^. In contrast, recordings from CA1 stratum radiatum as well as from the prefrontal cortex exhibited only theta-gamma, but no theta-fast gamma coupling irrespective of behavioral state. Moreover, prefrontal theta-gamma coupling was much weaker in REM sleep than during aWk. These observations in wild-type animals are in accordance with previous studies demonstrating that the patterns of phase-amplitude coupling depend on the recorded brain region as well as behavioral state^13^,^26^,^27^,^28^,^34^,^35^,^47^. In addition, we showed that APP is necessary for theta phase modulation of both gamma and fast-gamma. While the biophysical origins of fast-gamma oscillations remain unknown^35^, this result suggests that the network mechanisms underlying theta-gamma and theta-fast gamma coupling may be similar.

Interneuron networks are believed to take part in the generation of not only gamma but also theta oscillations^48^,^49^. Moreover, recent *in vivo* findings indicate that synaptic inhibition onto parvalbumin-positive basket cells is required for theta-gamma CFC^50^. Therefore, local inhibition seems to participate in theta and gamma oscillations as well as in theta-gamma coupling. One potential explanation for our findings is that APP would affect theta-gamma coupling by exerting influence on local inhibitory networks. This hypothesis is supported by the recent finding that APP is highly expressed in parvalbumin-positive basket cells and essential to regulate tonic and phasic GABAergic transmission in the dentate gyrus^51^. For instance, APP-KO mice show strong reduction of ambient GABA release from interneurons, which leads to a decrease in tonic GABAergic inhibition in the dentate gyrus^51^. However, how APP can selectively disrupt CFC despite not altering the power of oscillations – that is, without affecting their genesis – remains to be understood.

It should be noted that theta oscillations in the parietal cortex have been shown to be volume-conducted from the hippocampus, while parietal gamma oscillations would be generated locally^40^,^45^. Consistent with this, in one additional control animal we found that the amplitude of parietal theta oscillations was drastically reduced when a local electrode was used as reference, while the power peaks at gamma and fast gamma bands persisted with the local referencing (Supplementary Fig. S4). Thus, the theta-gamma coupling observed in the parietal cortex actually corresponds to the coupling between hippocampal-generated theta and parietal gamma oscillations^26^ (see also ref. 40). Indeed, in our data parietal theta was highly coherent with hippocampal theta, and cross-regional CFC between the hippocampus and parietal cortex led to identical results as the CFC analysis performed within these regions (not shown). On the other hand, theta coherence between the hippocampus and lateral PFC was much lower, and cross-regional CFC between the latter regions differed from local CFC. Nevertheless, there has been some controversy over whether theta oscillations recorded in the PFC are locally generated or volume-conducted from the hippocampus (or a mix of both), with evidence for^40^ and against^52^ volume conduction.

Gamma oscillations in PFC have also been shown to phase-lock to hippocampal theta in behaving rodents^40^. In contrast, here we found in control animals that gamma amplitude in the lateral PFC was at best only weakly coupled to theta recorded in the dorsal hippocampus. Instead, our data showed that gamma oscillations in the dorsal hippocampus are modulated by theta recorded in the lateral PFC. While the discrepancy between ours and a previous study^40^ regarding PFC gamma modulation by hippocampal theta may be due to differences in data analysis and/or recording position (we recorded in the lateral and more deeper PFC layers than ref. 40), our finding of PFC theta modulating hippocampal gamma is consistent with the notion that frontal areas exert top-down influences in other brain regions by long-range projections^53^. In line with this idea, a recent study showed increased CFC between frontal theta phase and parietal/occipital gamma amplitude during the formation of new memories in human^54^. The disruption of this long-range phase-amplitude coupling in mice lacking APP could be a contributing factor to the cognitive deficits observed in spatial maze tasks^9^,^32^,^55^, and is consistent with the hypothesis that phase-amplitude coupling may facilitate information processing across brain regions^17^.

In summary, here we have shown that APP is critical for the interaction of LFP oscillations of different frequencies. Intriguingly, our findings reveal that APP is not necessary for the generation of field potential rhythms per se. These results suggest a dissociation between the neuronal circuitries that generate LFP oscillations and the network mechanisms underlying CFC. Importantly, as APP is specifically involved in impaired CFC, our work opens new perspectives to develop biomarkers for APP dysfunction in AD.

## Methods

### Mice

APP-KO mice were generated by breeding homozygous knockout mice as described before^56^. All mice had been backcrossed to C57BL/6 for at least 6 generations. Control mice were age-matched wild type C57BL/6. Nine controls and nine APP-KO mice (36 weeks) were housed in a room with a 12-h light/dark circadian cycle (lights on 19:00–7:00) and with *ad libitum* access to food and water. All procedures were performed in accordance with the European Science Foundation Policy on the Use of Animals in Research and have been approved by the Governmental Supervisory Panel on Animal Experiments of Baden Württemberg at Karlsruhe.

### Surgery and recording

Surgical and recording procedures were similar to previously described^26^. Briefly, anesthesia was induced with 4% isoflurane and maintained with 1.5–2% isoflurane in a stereotaxic frame. Lidocaine was locally injected in the incision site over the skull for local anesthesia; 0.1 mg/kg of buprenorphin was injected subcutaneously prior to and 8 h after surgery for analgesia. Body temperature was maintained at around 36–37 °C and monitored throughout surgery. For LFP recordings, custom made electrodes constructed from two varnish-insulated nichrome wires (100-μm diameter, glued together) with different depth were implanted into the right dorsal hippocampal CA1 area (AP: −2.5 mm, ML: −1.5 mm, DV: −2.0 mm from dura). A single nichrome wire was implanted into the right lateral PFC (AP: +2.95 mm, ML: −1.5 mm, DV: −0.75 mm from dura). In addition, one stainless steel screw over the left posterior parietal cortex was used for surface EEG recording (AP: −2.5 mm, ML: −1.5 mm). Two screws driven into the bone above the cerebellum served as reference and ground electrodes. After electrode implantation, animals were housed individually. After one week of recovery, recording sessions started with three habituation periods in the home cage (two hour recordings on the first day, three hours on the second day and four hours on the third day). We used a miniaturized data logger (Neurologger 2A) with an accelerometer^57^,^58^. After mice adapted to the weight of the recording device, we proceeded to continuously record electrical activity across different states of the sleep-awake cycle. Raw data was amplified 1000x, band pass filtered (1–700 Hz) and digitized at 1600 Hz. The accelerometer registered velocity changes in 3-D with maximal sampling rate of 1 kHz. At the end of the experiment, mice were given an intravenous injection of ketamine/xylazine (0.01 ml/mg Rompun; Pfizer Pharma GmbH). For localization of electrode tips in the dorsal hippocampus and lateral PFC, we applied electric current to produce a lesion. Animals were then transcardially perfused with phosphate-buffered saline and, subsequently, 4% paraformaldehyde. Brains were sliced at 50 μm using a vibratome (Vibratome Series 1500, Germany), and mounted on slides to verify probe position by Nissl staining.

### Data analysis

LFP data were imported into MATLAB (The Mathworks Inc., Natick, MA) using built-in and custom written routines. We analyzed 30-s long contiguous LFP recordings randomly selected within the two states of pronounced theta activity: active waking (aWk) and REM sleep. This epoch length was chosen because it is large enough to allow for reliable CFC calculations^47^ while still lying within some REM sleep episodes. The results were cross-checked by analyzing a second set of randomly selected LFP epochs within aWk and REM states, which rendered qualitatively similar findings (not shown). State detection was based on movement activity recorded by the accelerometer, along with analysis of theta activity. REM sleep and aWk states roughly corresponded to ~4–7% and ~9–14% of all recording time, respectively. Both states were identified by the presence of prominent theta oscillations; aWk was further separated from REM sleep based on movement activity. Of note, the latter was not different between genotypes (Supplementary Fig. S5). For detailed description of behavioral staging, see ref. 59. Power spectra were computed using the Welch method (4-s windows, 2-s overlap). Band power of theta, gamma and fast gamma was defined as the average power in the frequency range of 4–12 Hz, 40–100 Hz and 120–160 Hz, respectively. Phase coherence was computed as the squared cross-power spectral density divided by the auto power spectral density in each region, which assumes values between 0 and 1.

To access the intensity of phase-amplitude coupling, we used the modulation index (MI) described in ref. 47. In brief, the phases of theta were binned into eighteen 20° intervals and the mean amplitude of gamma or fast gamma was calculated in each phase bin. A phase-amplitude distribution was achieved by normalization of gamma amplitude in each phase bin by the sum of amplitude values across bins. In this framework, coupling between theta phase and gamma amplitude is absent if the mean gamma amplitude is uniformly distributed over the theta phases. The higher the coupling, the further away the phase-amplitude distribution is from the uniform distribution. The MI is a measure of divergence of the phase-amplitude distribution from the uniform distribution based on the Kullback-Leibler distance^47^, normalized to achieve values between 0 and 1. The comodulation map is obtained by computing the MI of several frequency band pairs and expressing the results in a two-dimensional pseudocolor plot. One of the dimensions indicates the frequency bands analyzed as phase-modulating and the other represents the frequency bands analyzed as amplitude-modulated. The frequency bands are narrow-filtered (phase frequencies: 4-Hz bandwidths; amplitude frequencies: 10-Hz bandwidths) and each coordinate in the comodulation map represents the center frequency. A warm color in the comodulation map indicates coupling between the phase of the frequency band in the x axis and the amplitude of the frequency band in the y axis. The mean theta-(fast) gamma MI refers to the average over all MI values that lie at the intersection of the theta and (fast) gamma bands in the comodulation map.

### Statistics

Normal distribution of data was validated with the Kolmogorov-Smirnov test. For group comparisons we used the unpaired t-test for normally distributed data and the Mann-Whitney test for non-normally distributed data. All data are shown as boxplots (bars represent 2.5; 25; 50; 75; 97.5%). Significance levels are indicated by one or two asterisks (*p < 0.05, **p < 0.01). Since theta-(fast) gamma coupling has been previously demonstrated^35^,^47^,^60^, in this work we focused on comparing coupling levels between wild-type ad KO animals. Nevertheless, we also performed surrogate analyses (we computed MI values for contiguous phase and amplitude time series not matched in time) and derived a conservative threshold of 2 × 10^−4^ (above all surrogate values) for CFC significance within animals.

## Additional Information

**How to cite this article**: Zhang, X. *et al.* Impaired theta-gamma coupling in APP-deficient mice. *Sci. Rep.* **6**, 21948; doi: 10.1038/srep21948 (2016).

## Supplementary Material

Supplementary Information
