# Supplementary material for: Impaired theta-gamma coupling in APP-deficient mice
Source: Sci Rep. 2016 Feb 24;6:21948. doi: 10.1038/srep21948 (PMC4764939; doi:10.1038/srep21948)
Supplement: Supplementary Information [file srep21948-s1.pdf]

Impaired theta-gamma coupling in APP-deficient mice

Xiaomin Zhang[1], Weiwei Zhong[1], Jurij Brankačk[1], Sascha W. Weyer[2], Ulrike C. Müller[2], Adriano B. L. Tort[3*], Andreas Draguhn[1*]

1: Institute for Physiology and Pathophysiology, Heidelberg University, Heidelberg, Germany
2: Institute of Pharmacy and Molecular Biotechnology, Department of Bioinformatics and Functional Genomics, Heidelberg University, Heidelberg, Germany
3: Brain Institute, Federal University of Rio Grande do Norte, Natal, Brazil

Supplementary Information

5 Supplementary Figures + Legends

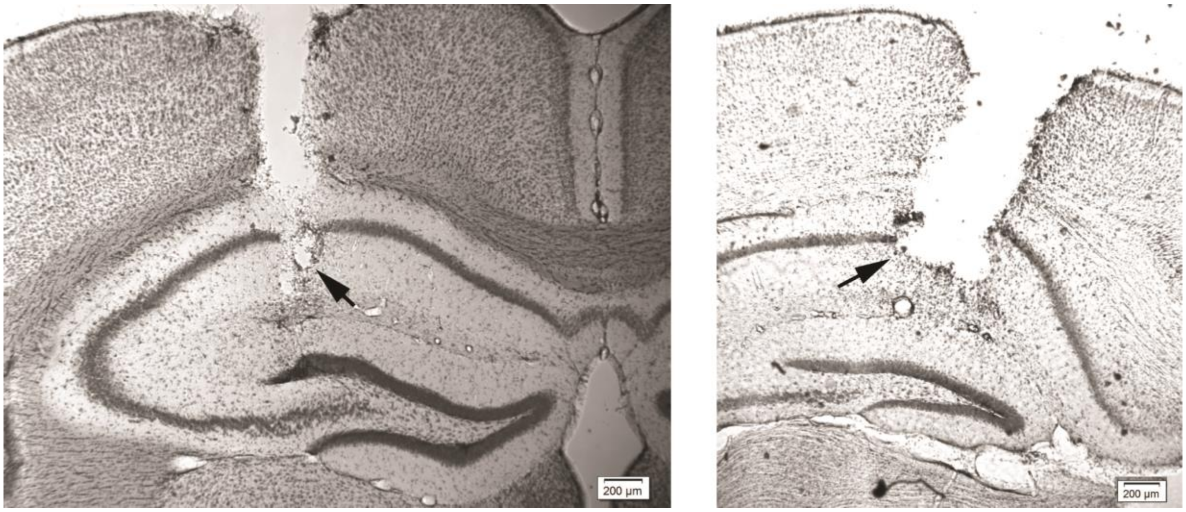

Supplementary Figure S1. Example of histological sections showing recording location in the hippocampus.

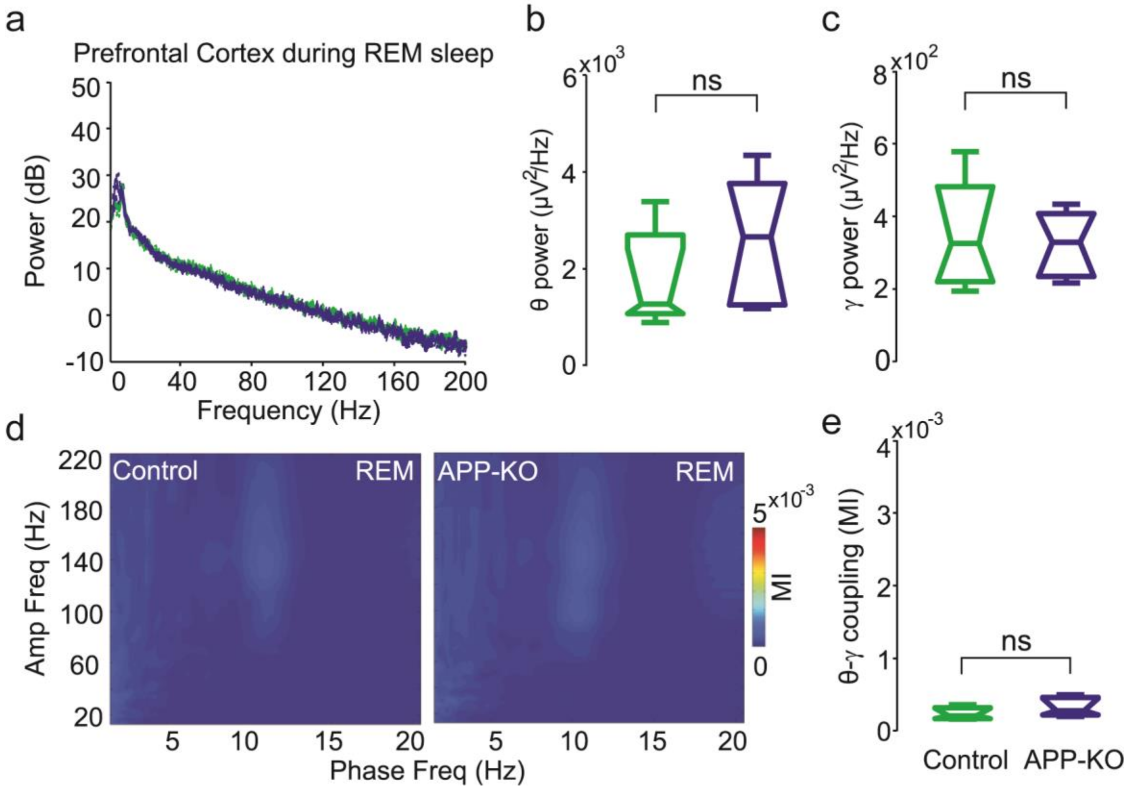

Supplementary Figure S2. Low cross-frequency coupling during REM sleep in recordings from the prefrontal cortex of both control and APP-KO mice. (a-e) Shown are mean power spectra (a), mean theta (b) and gamma (c) band power, mean comodulation map (d), and mean theta-gamma coupling strength (e) for PFC recordings during REM sleep in control (green) and APP-KO (blue) mice. Notice no difference in power content, along with low levels of CFC in both genotypes.

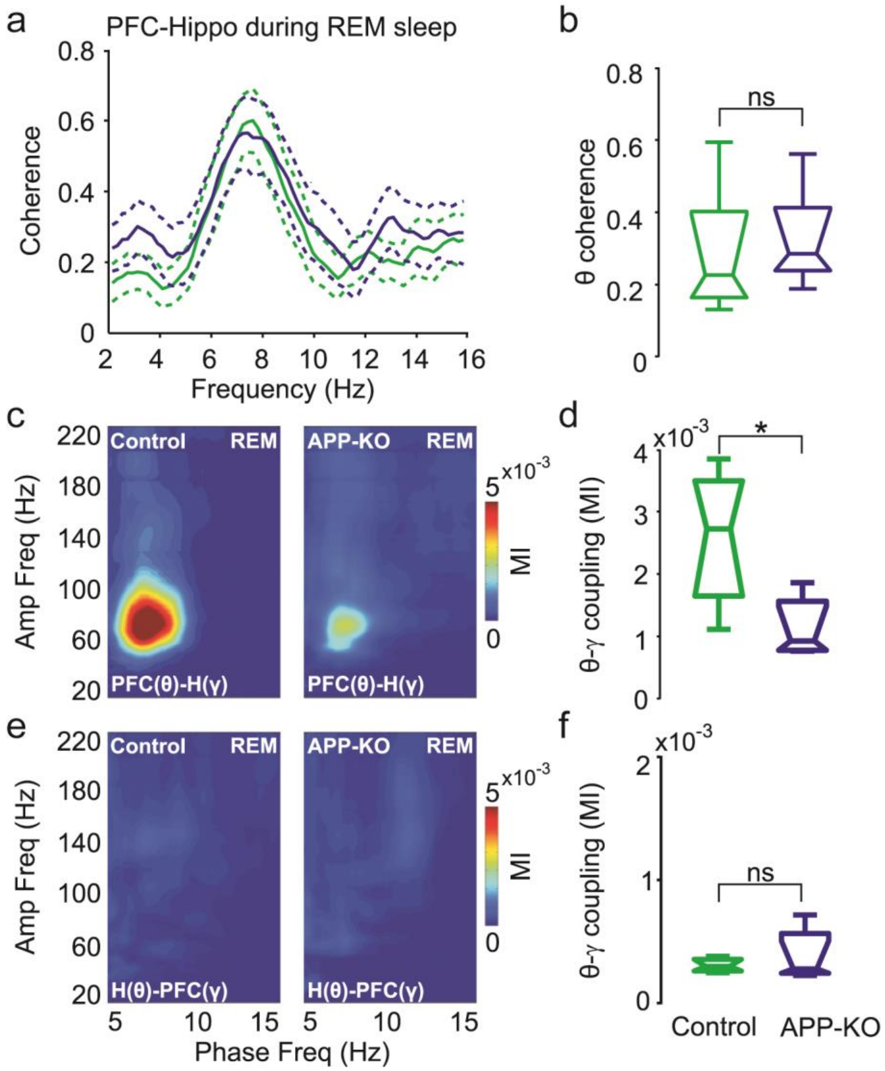

Supplementary Figure S3. Impaired cross-regional coupling between LFPs recorded from the dorsal hippocampus and lateral prefrontal cortex during REM sleep in APP-KO mice. (a) Phase coherence spectra between the hippocampus and PFC during REM sleep for control (green) and APP-KO (blue) mice. (b) Mean coherence in the theta band. Theta coherence is not different between control and APP-KO mice. (c) Mean cross-regional comodulation map obtained during REM using the instantaneous theta phase in the PFC and gamma amplitude in the hippocampus (PFC(θ)-H(γ)). (d) Mean PFC(θ)-H(γ) MI, showing impaired cross-regional coupling in APP-KO mice (*$p<0.05$). (e) Mean comodulation map for theta phase recorded in the hippocampus and gamma amplitude in the PFC (H(θ)-PFC(γ)) during REM sleep. (F) Mean H(θ)-PFC(γ) MI. Notice low CFC levels in both genotypes.

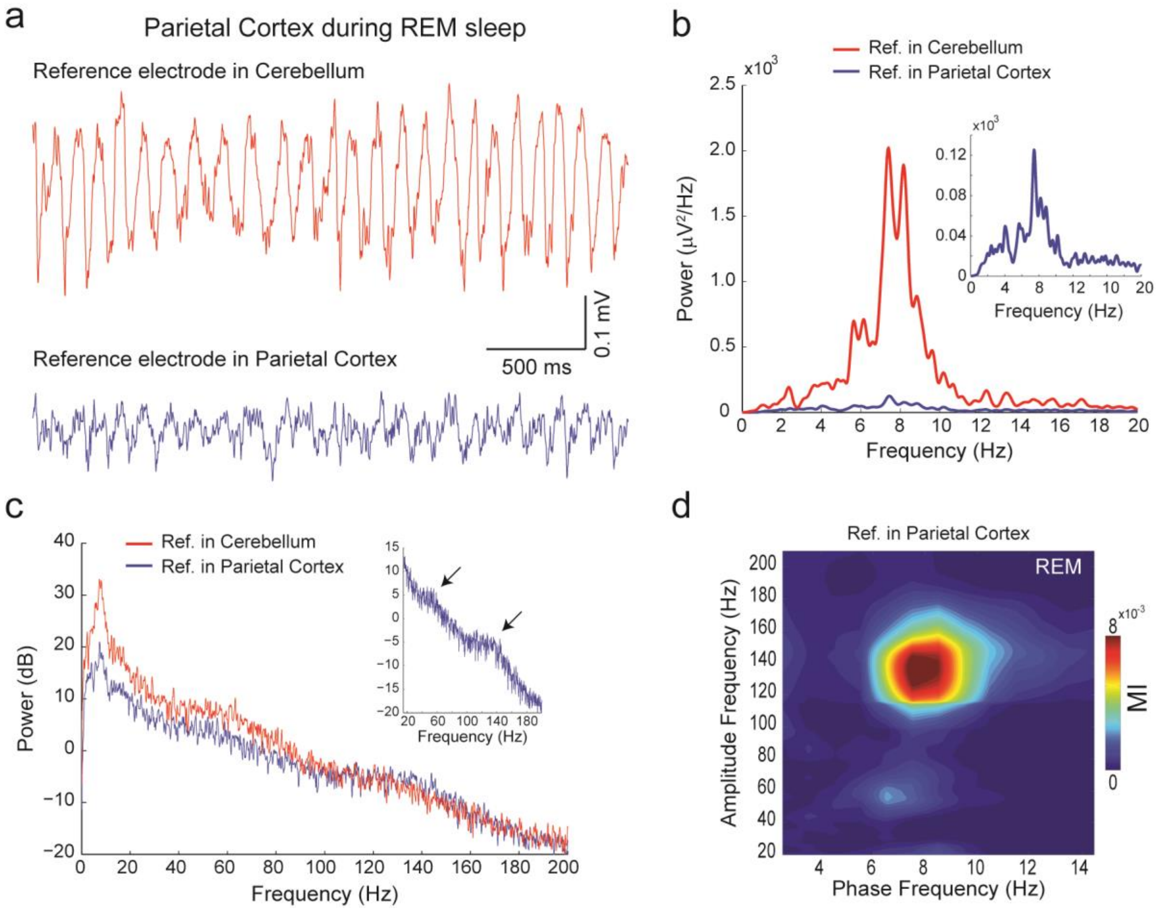

Supplementary Figure S4. Theta oscillations in the parietal cortex are volume-conducted from the hippocampus, while gamma and fast-gamma are generated locally. (a) Raw signals simultaneously recorded from the parietal cortex during REM sleep when the reference electrode is located at the skull above the cerebellum (red) or in the parietal cortex (blue). (b) Power spectra from 0 to 20 Hz. Inset shows that the local referencing still exhibits a power peak at theta. (c) Mean power spectra from 0 to 200 Hz in dB scale. The inset highlights that power bumps at gamma and fast gamma persist after the local referencing. (d) Comodulation map for the local referencing. Even though theta amplitude is drastically reduced, prominent theta-fast gamma coupling can still be observed during REM sleep since the MI only takes into account theta phase and not amplitude.

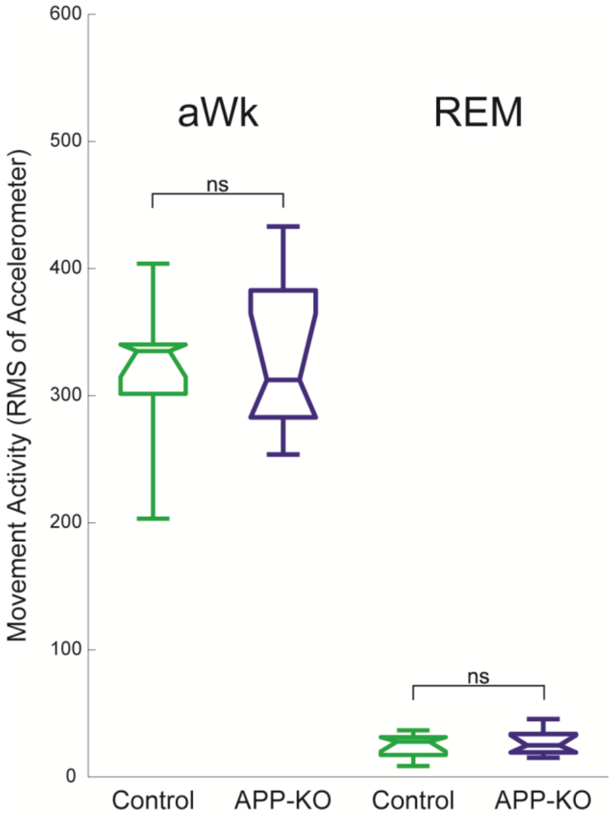

Supplementary Figure S5. Control and APP-KO mice display similar activity levels. Movement activity was estimated by the root mean square (RMS) of accelerometer signals and used to differentiate active waking (aWk) from REM sleep states.
